# Solving the limited availability of Astatine-211 from a European perspective: from production to the end user

**DOI:** 10.1186/s41181-026-00428-0

**Published:** 2026-02-17

**Authors:** Sture Lindegren, Holger Jensen, Hans Van de Maele, Renata Mikolajczak, Haingo Rabarijaona, Emma Aneheim

**Affiliations:** 1 Department of Medical Radiation Sciences, Institute of Clinical Sciences, Sahlgrenska Academy, University of Gothenburg, SE41345 Gothenburg, Sweden; 2 Department of Clinical Physiology and Nuclear Medicine, Cyclotron and Radiochemistry unit, Rigshospitalet, Denmark; 3 Radioisotope Centre POLATOM, National Centre for Nuclear Research, 05-400 Otwock, Poland; 4 ORI Consult/PHSE International, Brussels, Belgium; 5 Region Västra Götaland, Department of Oncology, Sahlgrenska University Hospital, SE41345 Gothenburg, Sweden

**Keywords:** Astatine-211, Production, Availability, Europe

## Abstract

**Background:**

Astatine-211, ^211^At, has long been a candidate for Targeted Alpha Therapy, TAT. However, over time, hurdles in the development of chemistry, the establishment of radiopharmacies, and the demonstration of its potential in clinical trials have been hampered by its limited availability. It is one of the rarest elements on earth and must be produced artificially. The main production route is by irradiating natural bismuth with helium ions in a cyclotron, utilizing the nuclear reaction ^209^Bi(α,2n)^211^At. It requires a medium-energy cyclotron capable of producing a 29 MeV α-beam. Early on, there were several such cyclotrons in Europe and worldwide, but to this day, only a few have been producing ^211^At. Now, many of the old cyclotrons have been decommissioned, leaving even fewer options. However, the situation is about to change with the installation of several new cyclotrons with the capacity to produce a relevant α-beam. In addition, there are also prospects evaluating the production of ^211^At in linear particle accelerators, LINACs, with which ^211^At potentially can be produced in very high amounts and high activity levels. Taking advantage of LINAC machines and new and old cyclotrons still in operation can solve the limited access to ^211^At today. With the production capacity in place, the astatine produced must be delivered in a relevant form to the end user. For this purpose, it also needs to meet all regulations for transporting radioactive material.

**Main body:**

This work is the result of European Cooperation in Science and Technology, COST Action CA 19114, Network for Optimized Astatine labeled Radiopharmaceuticals, NOAR, Work Group 1 assignments, focusing on all aspects on ^211^At production and availability. The review addresses the progress of ^211^At in terms of the requirement for its targetry, production, transport and the chemical and physical form for its delivery.

**Conclusion:**

With all efforts in production and making ^211^At available it has the potential to be the next generation Targeted Alpha Therapy radionuclide in Europe and worldwide.

## Background

Targeted Alpha Therapy, TAT, has emerged as a treatment strategy for disseminated cancer (Bruland et al. 2023; Iorio 2025; Group TATW 2018). In this field, astatine-211, ^211^At, is one of the most promising alpha-emitting radionuclides (Albertsson et al. 2022; Watabe et al. 2025; 2025; Hlongwa et al. 2025). However, ^211^At has suffered from a misconception that it is difficult or even impossible to produce in sufficient amounts for clinical applications (Mobina et al. 2023). The reason for this is the current lack of production units, i.e., lack of cyclotrons that can produce a relevant α-beam for the making of ^211^At (Vaidyanathan and Zalutsky 2008; Wilbur 2001). The main solution to this problem of astatine access and availability is increased investment in production capacity. However, a key to undertaking the relatively high investment cost required to establish a production network for ^211^At is that it must be proven in clinical trials (Zimmermann 2025). Several initial phase I clinical studies are in pipeline in Europe (and worldwide) investigating ^211^At-radiopharmaceuticals for different cancer indications (Müller et al. 2026). Those studies can be supplied with the production capacity of today.

However, to meet the demand of ^211^At for larger clinical trials, phase II-III studies, much higher amounts of ^211^At activity must be produced. Expected amounts required for a Phase II-III study would be > 100 GBq and if ^211^At ultimately is applied in general clinical practice the demand will be significantly higher. Therefore, a European production network would need to include not only investment in new dedicated ^211^At cyclotrons but also increased ^211^At production capacity using existing cyclotrons in operation and start-up of ^211^At production in operable cyclotrons capable of producing a α-beam. From this perspective, it should be noted that in a suitable cyclotron, ^211^At can be produced comparatively easily and straightforwardly (Feng and Zalutsky 2021; Lindegren et al. 2020, 2020; Zalutsky and Pruszynski 2011). The target material required to produce ^211^At is natural bismuth, which is abundant, safe to handle and cheap, which makes targets cost-effective and easy to manufacture. In comparison, target material for the production of other alpha-emitting radionuclides that are of interest for medical applications e.g., ^225^Ac, ^227^Th, and ^212^Pb, requires legacy or highly radioactive starting material for their production (Apostolidis et al. 2005; Radchenko et al. 2021). The targetry and the production of ^211^At are therefore much more convenient (Zalutsky and Pruszynski 2011; Matyskin et al. 2024).

One restriction of astatine production, though, is that there is a limitation in capacity at each cyclotron, which means that limited amounts of ^211^At can be produced per unit, and due to the relatively short half-life of ^211^At (t_1/2_=7,2 h), it therefore needs to be produced on demand. This requires several strategically located cyclotrons in operation to geographically cover the future need of this nuclide. However, the geographical distance to which ^211^At can be delivered is not only dependent on the half-life but also on the production capacity, the chemical form of ^211^At, transport logistics, and the end user resources and activity demand (Zimmermann 2025).

Efforts to overcome these hurdles with access and availability of ^211^At have been initiated, including both academic and commercial interests. Several cyclotron manufacturers either already produce or are developing new machines capable of producing intense α-beams to produce high amounts of ^211^At (Tosato et al. 2025).

Another way to address the limited capacity of ^211^At production is via linear particle accelerators, LINACs (Vretenar 2025; Ansari-Chauveau et al. 2025). These systems can further reduce access issues to ^211^At. They use the same nuclear reaction, the same target material, and beam energy as in cyclotron production, but unlike cyclotrons, LINAC systems have the potential to generate much higher beam currents on the target, leading to significantly higher activities of ^211^At produced. However, in LINAC production, the high beam current requires more complex target systems that can withstand the pulsed, high-intensity beams. Until these target challenges are resolved, cyclotrons will remain the primary source for ^211^At production.

After production, the astatine will be shipped to the end user and depending on the resources at the cyclotron site and at the receiving laboratory, as well as transportation times, several delivery forms of ^211^At are possible, ranging from the irradiated target to a finished drug product. Ultimately, a transnational delivery chain, meeting all legal requirements, must be set up to geographically cover the demand for this radionuclide.

## Main text

### Targetry

To produce ^211^At, bismuth targets are manufactured. In this process, high-purity natural bismuth is typically melted, vapor deposited, or electroplated onto a solid support (Schlyer and Ferrieri 2000). Although copper and ceramic material have been used as backing materials the support or backing is generally made from ultra-pure aluminum to prevent activation of impurities in the aluminum and to ensure high heat conductivity for effective target cooling during production (Larsen et al. 1996). The melting method results in a thicker initial layer of bismuth, which must then be milled or polished to achieve the optimal thickness (Henriksen et al. 2001). Compared to the melting technique, electroplating and vapor deposition techniques enable a significantly thinner and more uniform target layer of bismuth.

The aluminum backing material should be of high purity. A benchmark for aluminum quality would be ≥ 99.7% purity. Depending on the purity and the source of aluminum, impurities such as Li, Na, Mg, Si, Ca, Mn, Fe, and Cu can be activated during production of ^211^At. In Table 1 an example of co-produced radionuclides from Al impurities after ^211^At production, using the Scanditronix MC 32-NI cyclotron at Rigshospitalet, Copenhagen, Denmark, is shown. However, most of the co-produced isotopes have short half-lives and can be ignored. To avoid longer-lived radionuclides co-produced from aluminum impurities, a careful selection of backing material must be done, and in any case, the co-produced radionuclides must be separated from the astatine downstream in the isolation process.

**Table 1:** Astatine production and the main co-produced radionuclides seen at Rigshospitalet, Denmark

	Values end of bombardment	Half-life t_1/2_
Radionuclidic purity	At-211 ≥ 99.9%	7.214 h
Radionuclidic impurities	At-210 ≤ 0.04%	8.1 h
	Na-24 ≤ 0.02%	14.959 h
	Mg-27 ≤ 0.003%	9.458 m
	Al-29 ≤ 0.006%	6.56 m

Aluminum purity of the backing was 99.7%, and the applied α-beam energy was 29 MeV.

Although there are many advantages of bismuth as a target material for producing ^211^At, there are also some constraints related to its physical properties (Rabiei et al. 2024). The α-beam produces heat on the target, and bismuth has both low thermal conductivity and low heat capacity. The bismuth melting point is 271,4 C, with a conductivity of 7,87 W/(m⋅K), compared to 660,3 C° and 237 W/(m⋅K) for aluminum. It is therefore necessary to precisely control the Bi-layer thickness in the target manufacturing process. In terms of cooling the Bi-layer during ^211^At production, a thinner vapor-deposited layer of bismuth may be advantageous over thicker layers given the better thermal properties of aluminum compared to bismuth. To maximize the production of ^211^At, optimization between target cooling and applied beam current is needed. Grazing angle irradiations, with the beam hitting the target at 5–15 degrees, are measures often used to distribute the deposited heat over a larger target surface area and, in this way, reduce the target’s temperature. The better the cooling of the target is, the higher the current can be applied, and consequently, higher amounts of ^211^At can be produced.

There are two cyclotron configurations when producing ^211^At, an internal production or an external beam setup (Larsen et al. 1996; McIntosh et al. 2023). In internal production, the target is mounted on a probe with internal cooling on the backside of the target, normally water cooling. The probe is inserted inside the cyclotron to catch the α-beam at 28−29 MeV (Lindegren et al. 2001). In the external beam setup, the α-beam is extracted by a deflector system and guided via a beamline to a target station where it hits the target, generally with water cooling on the backside and helium cooling on the front of the target (Zalutsky and Pruszynski 2011).

### Production

Although several routes to produce ^211^At are possible, the main route is the transformation of natural bismuth via the nuclear reaction ^209^Bi(α,2n)^211^At. This can be achieved by a medium-energy cyclotron or in a linear accelerator capable of producing a 28−29 MeV α-beam.

**Fig. 1:**
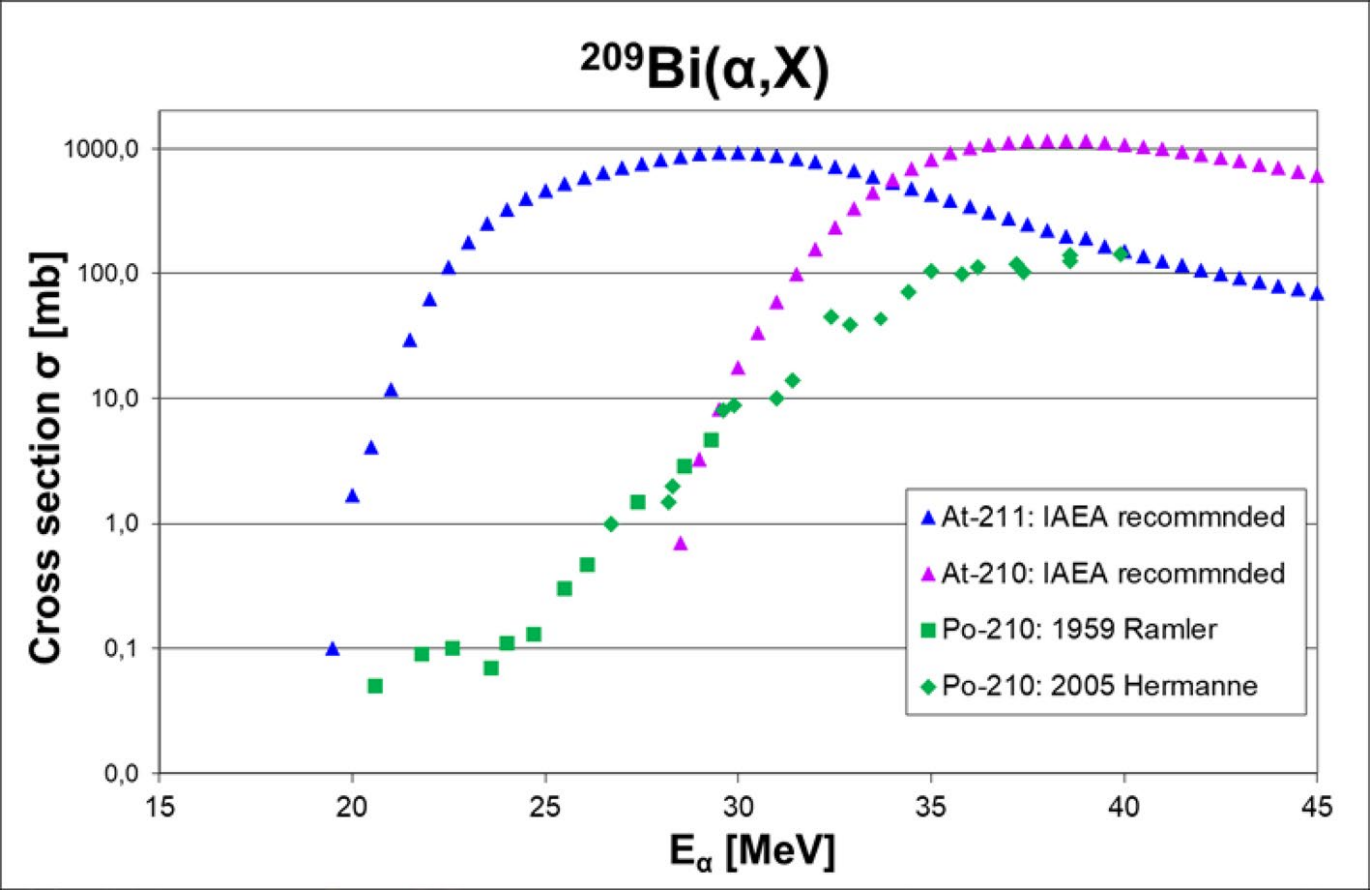
Cross sections for ^211^At, ^210^At, and ^210^Po produced by the nuclear reactions ^209^Bi(α,2n)^211^At, ^209^Bi (α,3n)^210^At and ^209^Bi(α,t)^210^Po, respectively

The optimal cross section for ^211^At production using the α,2n reaction is at around 31 MeV of the α-beam (Groppi et al. 2005). However, the beam energy needs to be kept at ≤ 29 MeV to minimize the coproduction of ^210^At, which decays to the unwanted toxic daughter ^210^Po (Ansari-Chauveau et al. 2025; Meyer and Astatine 2018; Schultz et al. 2006). Besides the unwanted ^209^Bi(α,3n)^210^At reaction, there is also co-production of an additional small amount of ^210^Po due to the cross section for the ^209^Bi(α,t)^210^Po reaction (Sevenois et al. 2023) (See Fig. 1). The direct production of ^210^Po is less problematic than the ^210^Po decay product from ^210^At, as directly produced ^210^Po can be separated from the desired ^211^At downstream in the workup process, either through dry distillation or wet extraction (Lindegren et al. 2001; Balkin et al. 2013). If the α-beam is kept below 29 MeV, the ^210^At to ^211^At ratio will, for a typical irradiation time, be below 0.04%, and the ^210^Po activity originating from the decay of ^210^At will, for a typical patient dose, be less than 10 Bq i.e. it will not cause any radiation risks. However, if very high activity levels of ^211^At can be produced, e.g., in a LINAC setup, it may be wise to lower the energy of the α-beam to the threshold of the α,3n reaction i.e., keeping the α-beam energy at ≤ 28 MeV particularly if the produced ^211^At are to be shipped long distances (Haddad et al. 2011). As described above, there may also be co-production of short-lived radioactive nuclides from impurities in the Al-backing material, therefore, before dismantling the activated target after EOB, it is preferably left to cool to allow the short-lived nuclides to decay.

**Fig. 2:**
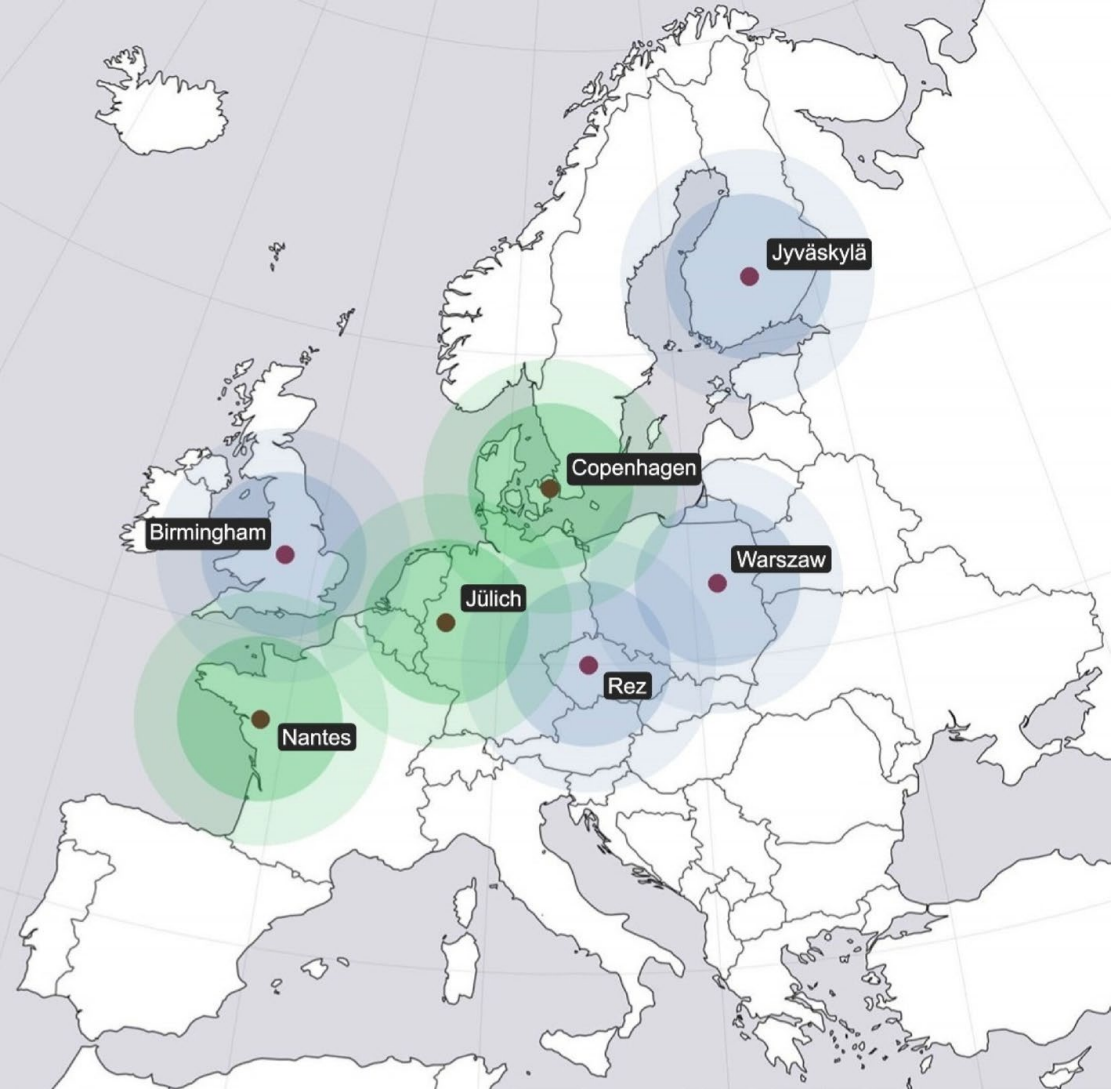
A schematic map over astaine-211 production sites in Europe: in regular operation (green) and small scale/to be started (blue). The inner and the outer circle correspond to 300 km and 450 km respectively

Today, only three sites in Europe are producing ^211^At regularly: the Scanditronix MC 32 cyclotron at the Cyclotron and Radiochemistry Unit, Copenhagen University Hospital, Copenhagen, Denmark (maximum production capacity at present: ca. 3 GBq at end of bombardment, EOB, per production, 5 productions / week), the IBA Cyclone^®^ 70 cyclotron at Arronax, Nantes, France (maximum production capacity at present: ca. 2 GBq EOB per production, 1 productions / week), and the IBA C30XP cyclotron in Jülich, Germany (maximum production capacity at present: ∼ 5.6 GBq EOB per production, 3–4 productions / week) (Lindegren et al. 2001; Haddad et al. 2011; Geets and Nactergal 2011). However, both new and existing cyclotrons will be added to the European ^211^At production network in the near future. In Warsaw, Poland, an IBA Cyclone^®^ C30XP has recently been installed, which will start optimizing the α-beam to produce astatine during 2026. An additional cyclotron in Europe that has confirmed to start up ^211^At production is the Isochronous Cyclotron U-120 M, in Rez, Czechia, which has produced astatine historically, and is now restarting production (Lebeda et al. 2005). Furthermore, the Scanditronix MC-40 cyclotron, in Birmingham, UK, can deliver a relevant α-beam and has recently initiated small-scale production of ^211^At. The Accelerator Laboratory at the University of Jyväskylä, Finland, has also shown interest in starting up ^211^At production and is expecting to produce an α-beam on target during 2025 (See Fig. 2).

Furthermore, new dedicated cyclotrons for the exclusive production of ^211^At are in the pipeline. Advanced Cyclotron Systems Inc., ACSI, Canada, has recently tested the applied current of their new ^211^At dedicated TR Alpha cyclotron. Sumitomo Heavy Industries Ltd, Japan, together with Osaka University in Japan, as well as IBA, Belgium, under the umbrella of the Innovative Health Initiative Joint Undertaking (IHI JU) grant, are both in the process of developing ^211^At-specific cyclotrons. Investment in these new powerful cyclotrons can change the limited supply dramatically. With a beam current of 200 µA and 4 h irradiation and 5 production days per week a production capacity of 500 GBq/week is expected for such a new production site. It should be noted, that for large scale productions of ^211^At it is important to optimize all production procedures to minimize radiation risk for the staff and correct waste handling. Ventilation and exhaust systems should be designed to trap possible release of ^211^At and other alpha emitters.

There is also a LINAC production of ^211^At under development, two in Europe at Grand Accelerateur National Dions, GANIL, Caen, France, and at the LINAC-4 project, CERN, Belgium, and two in the USA, at Nusano, West Valley City, UT, and at Colibri Isotopes, Lubbock, TX. When operational and after solving challenges around target design, these LINAC machines have the potential to produce high activities of ^211^At, e.g., Nusano will have the capacity to produce up to ∼1 × 10^6^ GBq EOB annually (personal communication). This means that LINAC production in the US could also be relevant from a European perspective if proper logistics and transport ways can be established.

### Waste management

When new highly efficient cyclotrons and LINAC machines have been taken int operation for ^211^At production the ^210^Po co-production through the nuclear reaction α,t will increase. Reported systemic burden health limit of ^210^ Po is < 0,2 Bq/kg-body-mass. Therefore, ^210^Po will be the main radionuclide for waste management. In addition, the ^210^At activity will also increase with increased production of ^211^At and as mentioned above the ^210^At/^211^At ratio should be low so the ^210^Po can be kept below the toxic limit for the patient. The ^210^At decay will also add to the ^210^Po waste particularly if the ^211^At e.g. the target is shipped long distances. Astatine-211 will also in 40% of the cases decay by α-emission to the beta emitter ^207^Bi (half-life 32 years) and will not pose any hazard for the patient as only ∼26 kBq^207^Bi/GBq will be produced from the ^211^At decay. Independent of the methods for isolating ^211^At from the irradiated target one must treat the backing or dissolved backing solutions as waste and, the chemistry solvent remains from the work up procedures and chemical development will also add to radioactive waste as it will contain the ^210^Po, and other impurities.

### Delivery forms of ^211^At and products thereof

After production, ^211^At is embedded in the solid bismuth layer of the target, and from there it must be converted into a chemically useful form. There are two general methods for isolating ^211^At from the irradiated bismuth target; dry distillation or wet extraction, where wet extraction can encompass both liquid-liquid extraction and solid phase extraction (McIntosh et al. 2023; Woen et al. 2020; Bourgeois et al. 2008; O’Hara et al. 2019). All three methods generally give high recovery yields. Following the isolation of ^211^At it is also important to determine radionuclide purity. Although most impurities are removed in the work up process breakthrough of impurities from production and from the bismuth backing material may occur (Tereshatov et al. 2023). In addition, ^210^At cannot be separated from ^211^At in the isolation process and will therefore be present in the subsequent chemistry and in a final radiopharmaceutical product and as mentioned above it is therefore important to keep the ^210^At/^211^At ratio low. For more extensive information on recovery of astatine from activated bismuth targets, we refer to the following previous reviews by Feng et al. (2021) and Lindegren et al. (2020). Independent of the methods used for isolating ^211^At from the irradiated target, the isolation procedure can be done either after production at the cyclotron site or at the end-user unit e.g., laboratory, hospital, radiopharmacy etc.

As the half-life of ^211^At is relatively short, 7.2 h, it should preferably be produced on demand and ideally in proximity to the recipient to optimize utilization efficiency. The form of ^211^At that can be received by the end user will depend on the resources at the cyclotron site, the resources at the final recipient unit and on the intended use of the activity e.g. basic chemistry, preclinical research, clinical applications, etc.

Delivery forms of ^211^At after production can be divided into non-processed or processed forms. Th non-processed form of ^211^At encompasses the irradiated target, in which the ^211^At is embedded in. As for the irradiated target as a delivery form, the cyclotron site only needs to manage production and, after End of Bombardment, EOB, dismantle, measure activity and pack the target for transport. The main advantage of using the target as the delivery form of ^211^At is that it will not be affected by radiolysis during transport, and, except for decay, no change in chemical or physical properties of the embedded ^211^At over time has been observed (Lindegren et al. 2001). Therefore, the target is well suited for both short and long-distance transport of ^211^At from the production site. However, this will also place special demands on the receiving lab, i.e., to have resources to isolate the ^211^At from the target material and the prospective work-up method, chemical synthesis, and/or formulation.

The simplest processed form of ^211^At for delivery is in a dry form after isolation from the target. This form of astatine can result from workup after dry distillation or astatine immobilized on a solid support, resin, following wet extraction (McIntosh et al. 2023; Balkin et al. 2013). However, recent work has shown that the reactivity of astatine in dry form after dry distillation decreases with the number of astatine decays e.g., during delivery, and hence the usability of such a form of astatine must be evaluated depending on the final application (Hansson et al. 2025). The stability over time of ^211^At isolated on solid support is yet to be reported. In cases where ^211^At is shipped in dry form, the isolation from the irradiated target, i.e., the distillation or wet extraction and dry preparation, is performed at or in proximity to the cyclotron site (Burns et al. 2020). Therefore, the recipient unit only needs resources for simple workup and chemistry, i.e., depending on the origin of the dry form of ^211^At, elution or dissolution, and the subsequent synthesis/formulation.

Another processed form of ^211^At for transportation is as a solute in aqueous or organic solvents. Water solvents will commonly relate to the astatide form, ^211^At^−^, which is present stabilized in, e.g., a strong basic solvent such as solutions of sodium hydroxide, NaOH, similarly to the common transport form of radioactive iodine, or dissolved in sodium hydrogen carbonate, NaHCO_3_ (Liu et al. 2020; Naka et al. 2024). Being a halogen, astatine is also readily soluble in several different organic solvents, where the most commonly used are chloroform and methanol (Aneheim et al. 2019; Pozzi and Zalutsky 2007). Chloroform is a good solvent for ^211^At and can be used to solvate the nuclide even at very high activity levels. Furthermore, the chloroform solvent can readily be evaporated to dryness with quantitative activity yields, leaving ^211^At as a dry residue (Aneheim et al. 2024). The dry ^211^At preparation allows for versatile subsequent chemistry (Hansson et al. 2025; Lindegren et al. 2008). Methanol may also be used to resolve ^211^At after isolation from the irradiated bismuth target, however, ^211^At is not fully stable in this solvent. Stabilization by introduction of e.g. N-chlorosuccinimide, NCS, is therefore recommended (Pozzi and Zalutsky 2017).

The most advanced processed form of ^211^At for shipment to the recipient lab/hospital would be the final ^211^At pharmaceutical product which can come in many different chemical formulations (Müller et al. 2026; Vanermen et al. 2024). In this case, the isolation from the target, the workup and the radiopharmaceutical synthesis should be performed near or in conjunction with the cyclotron. The transport of ^211^At pharmaceuticals will place high demands on the quality of the product and its formulation, which may have to include radical scavengers, e.g., ascorbate or gentisic acid, to improve stability.

In all cases where ^211^At is shipped in a solvated form, i.e., in water or organic solvents, as an intermediate compound or as a radiopharmaceutical, it should be noted that radiolysis may occur (Pozzi and Aneheim et al. 2019; Larsen and Bruland 1995; Ekberg et al. 2010). Even though the ^211^At may be stabilized by scavenger, etc (Zalutsky et al. 2001), it is recommended to consider only short-distance delivery for solvated ^211^At and/or ^211^At-compounds. Pros and cons of the different forms of astatine for delivery are summarized in Table 2.

**Table 2:** Delivery forms of ^211^At and the implications on radiochemical/technical demands on the production and recipient site as well as the potential shipping distance

Form of ^211^At for delivery	Demands on production site	Demands on recipient site	Radiolysis/Stability concerns	Suitable shipping distance
Irradiated Target	Low	High	Low/None	Long
Isolated dry form	Medium	Medium	Medium/High	Medium
Isolated wet form	Medium	Medium	Medium/High	Short
Radiopharmaceutical product	High	Low	High	Short

### Transport of ^211^At

**Fig. 3:**
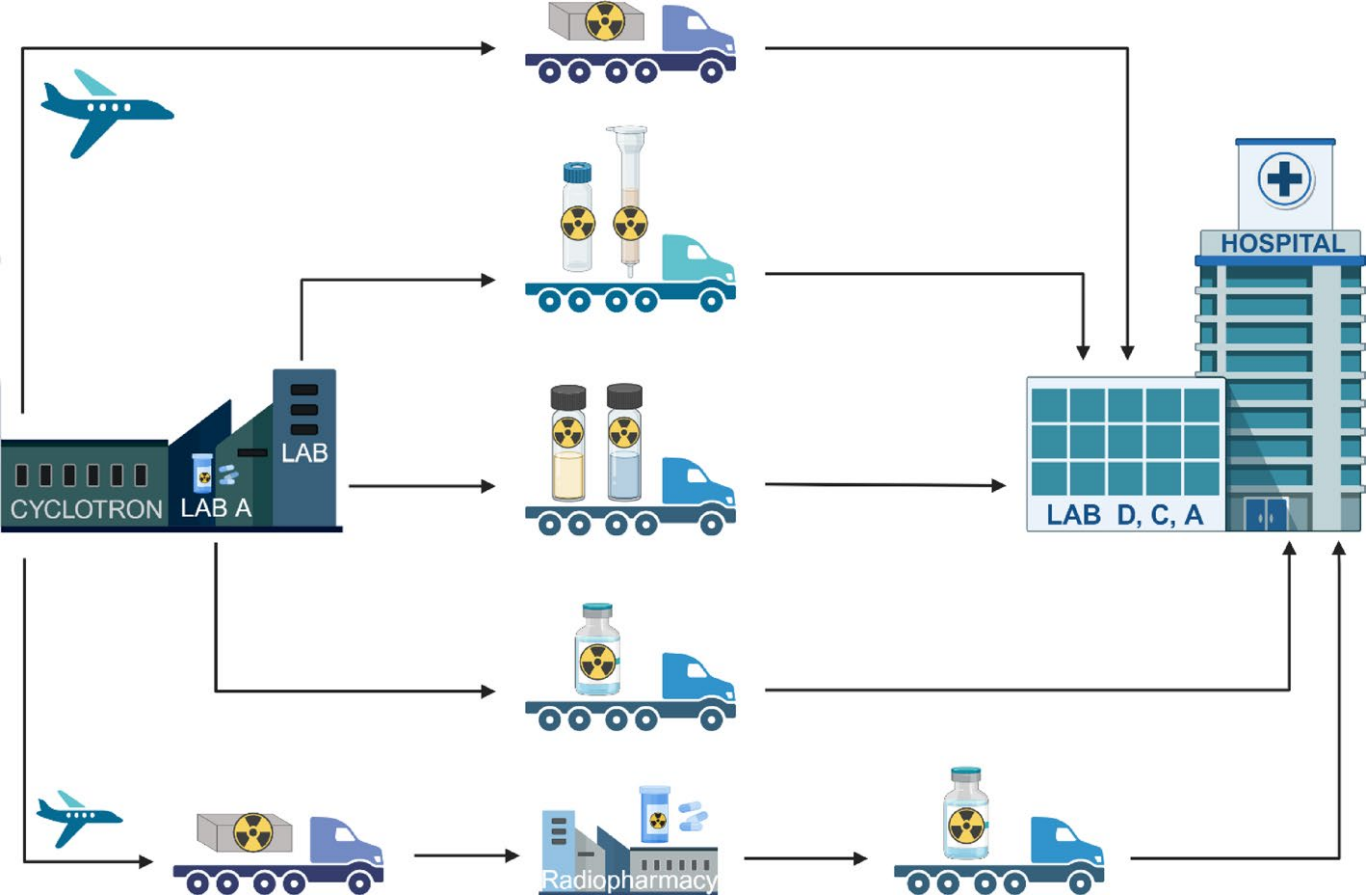
Overview of transport options of astatine-211 between the production facility and the end-user. The different forms of astatine-211 include the solid target, dry processed material, liquid processed material and the formulated pharmaceutical

The relatively short half-life of ^211^At, combined with complex radiological and logistical constraints, necessitates a specialized, regulated, and precisely coordinated transport process. Clinical treatment doses of ^211^At-pharmaceuticals typically range between 250 and 500 MBq per patient (Zimmermann 2025). Accounting for processing inefficiencies, activity losses during synthesis, and delays such as quality control, product release, and internal hospital transport, the required amount of ^211^At at the end of bombardment (EOB) may reach or exceed 1 GBq for a 300 MBq patient injection dose. These losses highlight the critical importance of time, as every hour post-EOB represents a significant loss in available activity. Including necessary target cooling, analysis, and packaging time, only a narrow window remains to transport the product to the end user before it loses clinical value. This time window, constrained by the short half-life of ^211^At, requires highly efficient logistics and infrastructure. Figure 2 depicts the current and near-future production sites in Europe, where the two circles around each site represent an expected driving distance of ca. 4 and 7 h. Such a 4 h transport is conducted weekly between the cyclotron in Copenhagen, Denmark, and the University of Gothenburg, Sweden, which historically has managed production for a clinical trial (Andersson et al. 2009). Taking the current production capacity of the cyclotrons and all factors mentioned above into account, the 7 h driving distance, roughly one half-life of At-211, would in many cases also allow for delivering a clinical dose of an At-211 radiopharmaceutical. The choice of transport mode (see Fig. 3) – road, air, or a combination – depends on the distance between the cyclotron and end-user facility, road quality, airport proximity, customs procedures, and available authorizations (Europe UNECf 2023). Due to the relatively short half-life of ^211^At air freight is often necessary for cross-border or long-distance delivery [Organization ICA 2003]. For shorter distances, road transport may suffice but must still adhere to national and international regulations for Class 7 radioactive goods.

^211^At is categorized as a Class 7 dangerous good and is typically transported under the designation “Radioactive material, Type A package, non-special form, non-fissile” (UN 2915). These shipments must comply with the IAEA’s Regulations for the Safe Transport of Radioactive Material (Agency IAE 2018) which are embedded into broader frameworks such as the UN Orange Book and incorporated into the ADR for road transport in Europe, International Air Transport Association, IATA, for air freight, and respective national regulations (Europe UNECf 2023, Agency IAE 2018). In the case of ^211^At, which can be shipped as a solid target form or as a radiolabeled pharmaceutical in liquid form, the packaging approach may vary. Solid irradiated targets are typically shipped in Type A packages with shielding appropriate for low-energy gamma photons and characteristic X-rays from ^211^Po decay. Liquid forms of ^211^At, especially in radiopharmaceutical formulations, require enhanced containment measures to prevent leakage or contamination, while still fitting within Type A specifications.

Under the current IAEA SSR-6 Rev. 1 standard, the A2 limit for ^211^At is set at 0.5 TBq (500 GBq), allowing most radiopharmaceutical transport operations to use Type A packaging (Agency IAE 2018). However, in the revised SSR-6 Rev. 2, which is set to become effective in 2029, the A2 value for ^211^At will be reduced by more than two orders of magnitude, to just 0.004 TBq (4 GBq) (IAEA 2025). While this change will not take effect immediately, the ten-year transition period provides a critical window for stakeholders to assess and adapt their logistics strategies to Type B(U) packaging for higher activities of ^211^At i.e. >4 GBq, accordingly.

## Conclusions

Regarding the current limited access to ^211^At, this review highlights that targetry and cyclotron production per se is not a problem. Bismuth targetry is safe and cost-effective, and cyclotron production is comparatively straightforward. Therefore, with the high availability of bismuth, one can foresee a future of infinite production capacity of astatine. However, despite the relative ease of production of ^211^At, there is currently a limited number of operational medium-energy cyclotrons in Europe that can produce a relevant ∼30 MeV α-beam. These cyclotrons have the capacity to facilitate clinical trials with ^211^At in certain strategic locations already today, but for the greater Europe, only small-scale clinical research amounts are available at present. Regardless of the present shortage of producing capacity, there is an increasing academic and commercial interest in Targeted Alpha Therapy with ^211^At. With this new increased interest, many efforts have been made and are being initiated to increase availability. Most important is the development of new dedicated ^211^At cyclotrons, i.e., machines with a fixed ∼30 MeV alpha beam energy, including several cyclotron manufacturers. In this effort, the Canadian company Advanced Cyclotron Systems Inc. ACSI has developed a new machine, the TR-Alpha cyclotron, for large-scale production of ^211^At, which has been installed in China by Alpha Nuclide in early 2025. In Europe IBA, Belgium, is working with Framatome, France, creating a network of ^211^At production, including the development of a dedicated 30 MeV alpha cyclotron. Installation of new astatine-dedicated cyclotrons in Europe is therefore feasible but will be dependent on strategic investments and a further increased commercial interest in ^211^At pharmaceuticals. Besides the new cyclotrons in pipeline, there are a few available operable cyclotrons that will be added to the European network of ^211^At production in the near future (Fig. 2), increasing its availability. Another important production route to increase the future availability of ^211^At can also be realized by linear accelerators, GANIL, Caen, France, LINAC-4 project CERN, Belgium, or Nusano, UT, USA (Vretenar 2025, Ansari-Chauveau et al. 2025). The area/distance to which ^211^At can be delivered is related to the production capacity of the nuclide. Zimmerman suggests a 10 h decay range (Zimmermann 2025), which in a European perspective would allow road delivery from e.g. Nantes, France, to Barcelona, Spain, from Rez, Czech Republik, to Milan, Italy, or from Warsaw, Poland, to Budapest, Hungary. Starting with a, today achievable, amount of ^211^At of 3 GBq EOB, this means that ca. 1.1 GBq will remain after 10 h transport, enough for producing a clinical dose. If we consider 30 GBq of ^211^At at EOB, from a new dedicated At-211 cyclotron, then the distance may be increased way beyond the 10 h decay, covering most of Europe by plane or road, or allow for production of multiple patient doses closer to the production site. With LINAC production in place, the distance can be increased dramatically e.g. >24 h transport time would be reasonable. Successful transport would of course also relate to what form of ^211^At that is shipped to the end user, where the irradiated solid target would be the safest option for long-distance shipping. To further motivate investment in new production facilities and large-scale production of astatine, its efficacy needs to be proven in clinical applications. Although several clinical applications are planned, there have to this day only been two completed clinical trials (Albertsson et al. 2022; Zalutsky et al. 2008). However, in Japan, several clinical studies are ongoing, and many more studies are planned in Europe and worldwide (Watabe et al. 2025, 2025). The field of targeted alpha therapy with ^211^At is still emerging including the development of chemistry (Müller et al. 2026) and radiopharmacy to meet the increasing future demand and its use for clinical applications. For this purpose, a complete value chain needs to be created, including targetry, production network, delivery forms, transport, workup and radiopharmacy. The transport of ^211^At requires a regulated, carefully devised process that balances radiological safety, legal compliance, and timely delivery. As production and clinical use will scale up, logistics systems must evolve in parallel to ensure this promising therapeutic radionuclide reaches patients with both efficacy and safety preserved. Currently, logistics operators and health institutions must adapt existing Class 7 frameworks. Because of the tight window and growing clinical demand, early planning, cross-border regulatory harmonization, and high-performance logistics execution will be essential for the safe and timely delivery of ^211^At-based radiopharmaceuticals (Europe UNECf 2023, Agency IAE 2015, Agency IAE 2014). Several joint efforts, both academic and commercial initiatives, to solve the logistics and availability of ^211^At have been undertaken. A program focusing on all aspects of ^211^At has been supported in a European Cooperation in Science and Technology, COST Action CA 19,114, Network for Optimized Astatine labeled Radiopharmaceuticals (NOAR), including more than 20 European countries (NOAR 2024). This program ended late 2024, and from which a new organization, the World Astatine Community, WAC, has emerged, formed by representatives from the United States, Japan, and the European Union to share astatine production technology and advance science and health care (World Astatine Community 2023). PRISMAP – the European medical radionuclides programme, aimed to provide immediate access to novel radionuclides and to facilities where they can be readily used to support the ongoing research across Europe and beyond (Prismap Medical Radionuclides 2025). It has included ^211^At in its radionuclide portfolio (PRISMAM 2025) and has been offered to the PRISMAP user’s projects. At present, astatine research has also been recognized by a European Union funding opportunity, under the Innovative Health Initiative Joint Undertaking (IHI JU), where two consortia were funded, ACCELERATE.EU and THERA4CARE. The focus with the ACCELERATE.EU grant (Accelerate.EU 2025) is to establish a cross-EU supply chain of ^211^At, including the development of a new cyclotron for the specific production of this radionuclide. This program is also pioneering the development of ^211^At in personalized medicine to target aggressive cancers like pancreatic, breast, and brain cancer. THERA4CARE has a similar aim, focusing on developing and providing personalized, targeted theranostic radionuclide therapies, including several different radionuclides and ^211^At (Thera4Care 2025).

In summary, a prerequisite to solve hurdles with ^211^At (concerning chemistry, to establish radiopharmacies and showing its potential in clinical applications) is its production and availability, and one can conclude that with current academic and commercial attention, the access to ^211^At will increase significantly. With all these efforts, ^211^At has the potential to be the next generation Targeted Alpha Therapy radionuclide in Europe and worldwide.

## Data Availability

Not applicable.

## Abbreviations

LINAC: Linear accelerator
COST: European Cooperation in Science and Technology
NOAR: Network for Optimized Astatine labeled Radiopharmaceuticals
IHI JU: Innovative Health Initiative Joint Undertaking
EOB: End of Bombardment
WAC: World Astatine Community
PRISMAP: The European medical radionuclides programme

## References

[CR1] Accelerate EU. Elevating the future of cancer care with alpha theranostics. Innovative Health Initiative; 2025.

[CR2] Agency IAE. Radiation Protection and Safety of Radiation Sources — International Basic Safety Standards. GSR Part3 ed. Vienna; 2014.

[CR3] Agency IAE. Safety Guide on Packaging and Transport of Radioactive Material. Vienna; 2015.

[CR4] Agency IAE. Regulations for the Safe Transport of Radioactive Material. SSR-6 Rev.1 ed. Vienna; 2018.

[CR5] Albertsson P, Back T, Bergmark K, Hallqvist A, Johansson M, Aneheim E, et al. Astatine-211 based radionuclide therapy: current clinical trial landscape. Front Med (Lausanne). 2022;9:1076210. 10.3389/fmed.2022.1076210. 36687417 10.3389/fmed.2022.1076210 PMC9859440

[CR6] Andersson H, Cederkrantz E, Back T, Divgi C, Elgqvist J, Himmelman J, et al. Intraperitoneal alpha-particle radioimmunotherapy of ovarian cancer patients: pharmacokinetics and dosimetry of (211)At-MX35 F(ab’)2–a phase I study. J Nucl Med. 2009;50:1153–60. 10.2967/jnumed.109.062604. 19525452 10.2967/jnumed.109.062604

[CR7] Aneheim E, Hansson E, Timperanza C, Jensen H, Lindegren S. Behaviour, use and safety aspects of astatine-211 solvated in chloroform after dry distillation recovery. Sci Rep. 2024;14:9698. 10.1038/s41598-024-60615-4. 38678056 10.1038/s41598-024-60615-4 PMC11055885

[CR8] Aneheim E, Palm S, Jensen H, Ekberg C, Albertsson P, Lindegren S. Towards elucidating the radiochemistry of astatine - Behavior in chloroform. Sci Rep. 2019;9:15900. 10.1038/s41598-019-52365-5. 31685874 10.1038/s41598-019-52365-5 PMC6828679

[CR9] Ansari-Chauveau S, Frelin AM, de France G, Guertin A, Haddad F, Ledoux X, et al. Optimizing (211)At production cross section by studying the rise of (210)At cross section: first measurement using Linac SPIRAL2. Appl Radiat Isot. 2025;225:112061. 10.1016/j.apradiso.2025.112061. 40779853 10.1016/j.apradiso.2025.112061

[CR10] Apostolidis C, Molinet R, McGinley J, Abbas K, Mollenbeck J, Morgenstern A. Cyclotron production of Ac-225 for targeted alpha therapy. Appl Radiat Isot. 2005;62:383–7. 10.1016/j.apradiso.2004.06.013. 15607913 10.1016/j.apradiso.2004.06.013

[CR11] Balkin ER, Hamlin DK, Gagnon K, Chyan MK, Pal S, Watanabe S, et al. Evaluation of a wet chemistry method for isolation of cyclotron produced [At-211]Astatine. Appl Sci-Basel. 2013;3:636–55. 10.3390/app3030636.

[CR12] Bourgeois M, Guerard F, Alliot C, Mougin-Degraef M, Rajerison H, Remaud-Le Saec P, et al. Feasibility of the radioastatination of a monoclonal antibody with astatine-211 purified by wet extraction. J Label Comp Radiopharm. 2008;51:379–83. 10.1002/jlcr.1543. 10.1002/jlcr.1543 26148336

[CR13] Bruland OS, Larsen RH, Baum RP, Juzeniene A, Editorial. Targeted alpha particle therapy in oncology. Front Med (Lausanne). 2023;10:1165747. 10.3389/fmed.2023.1165747. 36960341 10.3389/fmed.2023.1165747 PMC10029265

[CR14] Burns JD, Tereshatov EE, McCarthy MA, McIntosh LA, Tabacaru GC, Yang X, et al. Astatine partitioning between nitric acid and conventional solvents: indication of covalency in ketone complexation of AtO+. Chem Commun (Camb). 2020;56:9004–7. 10.1039/d0cc03804k. 32638758 10.1039/d0cc03804k

[CR15] Ekberg C, Aneheim E, Fermvik A, Skarnemark G. Using 211 At as internal alpha radiolysis source allowing for simple detection of radiolysis products. Radiat Phys Chem. 2010;79:454–6. 10.1016/j.radphyschem.2009.10.003.

[CR16] Europe UNECf. European Agreement concerning the International Carriage of Dangerous Goods by Road(ADR). Geneva; 2023.

[CR17] Feng Y, Zalutsky MR. Production, purification and availability of (211)At: near term steps towards global access. Nucl Med Biol. 2021;100–101:12–23. 10.1016/j.nucmedbio.2021.05.007. 34144505 10.1016/j.nucmedbio.2021.05.007 PMC8448941

[CR18] Geets J-M, Nactergal B. The cyclone 30XP multiparticles cyclotron and At-211, Cu-67 production. J Nucl Med. 2011;52:1514.

[CR19] Groppi F, Bonardi ML, Birattari C, Menapace E, Abbas K, Holzwarth U, et al. Optimisation study of alpha-cyclotron production of At-211/Po-211 g for high-LET metabolic radiotherapy purposes. Appl Radiat Isot. 2005;63:621–31. 10.1016/j.apradiso.2005.05.041. 16055338 10.1016/j.apradiso.2005.05.041

[CR20] Group TATW. Targeted alpha Therapy, an emerging class of cancer agents: A review. JAMA Oncol. 2018;4:1765–72. 10.1001/jamaoncol.2018.4044. 30326033 10.1001/jamaoncol.2018.4044

[CR21] Haddad F, Barbet J, Chatal JF. The ARRONAX project. Curr Radiopharm. 2011;4:186–96. 10.2174/1874471011104030186. 22201708 10.2174/1874471011104030186

[CR22] Hansson E, Timperanza C, Jensen H, Eriksson B, Lindegren S, Aneheim E. Radiolabeling yield dependency of post-dry distillation decay of astatine-211. Nucl Med Biol. 2025;144–145:108999. 10.1016/j.nucmedbio.2025.108999. 39961172 10.1016/j.nucmedbio.2025.108999

[CR23] Henriksen G, Messelt S, Olsen E, Larsen RH. Optimisation of cyclotron production parameters for the 209Bi(alpha, 2n) 211At reaction related to biomedical use of 211At. Appl Radiat Isot. 2001;54:839–44. 10.1016/s0969-8043(00)00346-8. 11258534 10.1016/s0969-8043(00)00346-8

[CR24] Hermanne A, Tárkányi F, Takács S, Szücs Z, Shubin YN, Dityuk AI. Experimental study of the cross-sections of α-particle induced reactions on 209 Bi. Appl Radiat Isot. 2005;63:1–9. 10.1016/j.apradiso.2005.01.015. 15866442 10.1016/j.apradiso.2005.01.015

[CR25] Hlongwa KN, Rivombo PM, More SS. The advent of Astatine-211 in targeted radionuclide therapy in prostate cancer: will it come to true fruition? Q J Nucl Med Mol Imaging. 2025;69:180–5. 10.23736/S1824-4785.25.03643-X. 40605660 10.23736/S1824-4785.25.03643-X

[CR26] IAEA. Regulations for the Safe Transport of Radioactive Material. 2025 Edition.; 2025.

[CR27] Iorio Di V, Sarnelli A, Boschi S, Sansovini M, Genovese RM, Stefanescu C et al Recommendations on the clinical application and future potential of alpha-Particle therapy: A comprehensive review of the results from the SECURE project. Pharmaceuticals (Basel). 2025;18:1578. 10.3390/ph18101578. 41155690 10.3390/ph18101578 PMC12567000

[CR28] Larsen RH, Bruland OS. Radiolysis of Radioimmunoconjugates - Reduction in Antigen-Binding ability by Alpha-Particle radiation. J Label Compd Rad. 1995;36:1009–18. 10.1002/jlcr.2580361012.

[CR29] Larsen RH, Wieland BW, Zalutsky MR. Evaluation of an internal cyclotron target for the production of At-211 via the Bi-209 (alpha,2n)At-211 reaction. Appl Radiat Isot. 1996;47:135–43. doi:Doi 10.1016/0969–8043(95)00285-5. 8852627 10.1016/0969-8043(95)00285-5

[CR30] Larsen RH, Wieland BW, Zalutsky MR. Evaluation of an internal cyclotron target for the production of 211At via the 209Bi (alpha,2n)211 at reaction. Appl Radiat Isot. 1996;47:135–43. 8852627 10.1016/0969-8043(95)00285-5

[CR31] Lebeda O, Jiran R, Ralis J, Stursa J. A new internal target system for production of (211)At on the cyclotron U-120 M. Appl Radiat Isot. 2005;63:49–53. 10.1016/j.apradiso.2005.02.006. 15866447 10.1016/j.apradiso.2005.02.006

[CR32] Lindegren S, Albertsson P, Back T, Jensen H, Palm S, Aneheim E. Realizing clinical trials with Astatine-211: the chemistry infrastructure. Cancer Biother Radiopharm. 2020;35:425–36. 10.1089/cbr.2019.3055. 32077749 10.1089/cbr.2019.3055 PMC7465635

[CR33] Lindegren S, Back T, Jensen HJ. Dry-distillation of astatine-211 from irradiated bismuth targets: a time-saving procedure with high recovery yields. Appl Radiat Isot. 2001;55:157–60. 10.1016/s0969-8043(01)00044-6. 11393754 10.1016/s0969-8043(01)00044-6

[CR34] Lindegren S, Frost S, Back T, Haglund E, Elgqvist J, Jensen H. Direct procedure for the production of 211At-labeled antibodies with an epsilon-lysyl-3-(trimethylstannyl)benzamide Immunoconjugate. J Nucl Med. 2008;49:1537–45. 10.2967/jnumed.107.049833. 18703591 10.2967/jnumed.107.049833

[CR35] Liu Y, Watabe T, Kaneda-Nakashima K, Ooe K, Shirakami Y, Toyoshima A, et al. Preclinical evaluation of Radiation-Induced toxicity in targeted alpha therapy using [(211)At] NaAt in mice: A revisit. Transl Oncol. 2020;13:100757. 10.1016/j.tranon.2020.100757. 32220762 10.1016/j.tranon.2020.100757 PMC7109464

[CR36] Matyskin AV, Angermeier SB, Drera SS, Prible MC, Geuther JA, Heibel MD. Actinium-225 photonuclear production in nuclear reactors using a mixed radium-226 and gadolinium-157 target. Nucl Med Biol. 2024;136–137:108940. 10.1016/j.nucmedbio.2024.108940. 39002498 10.1016/j.nucmedbio.2024.108940

[CR37] McIntosh LA, Burns JD, Tereshatov EE, Muzzioli R, Hagel K, Jinadu NA, et al. Production, isolation, and shipment of clinically relevant quantities of astatine-211: a simple and efficient approach to increasing supply. Nucl Med Biol. 2023;126–127:108387. 10.1016/j.nucmedbio.2023.108387. 37837782 10.1016/j.nucmedbio.2023.108387

[CR38] Meyer GJ, Astatine. J Label Comp Radiopharm. 2018;61:154–64. 10.1002/jlcr.3573. 10.1002/jlcr.3573 29080397

[CR39] Mobina R, Vaez Alaei AR, Yousefnia H. Status of alpha-emitter radioimmunoconjugates for targeted therapy. Curr Radiopharm. 2023;16:85–94. 10.2174/1874471016666230106111119. 36627787 10.2174/1874471016666230106111119

[CR40] Müller M, Pedersen NB, Shalgunov V, Jensen AI, Battisti UM, Herth MM. Astatine-211-Towards in vivo stable Astatine-211 labeled radiopharmaceuticals and their (Pre)Clinical applications. Med Res Rev. 2026;46:203–37. 10.1002/med.70008. 40888104 10.1002/med.70008 PMC12673465

[CR41] Naka S, Ooe K, Shirakami Y, Kurimoto K, Sakai T, Takahashi K, et al. Production of [(211)At]NaAt solution under GMP compliance for investigator-initiated clinical trial. EJNMMI Radiopharm Chem. 2024;9:29. 10.1186/s41181-024-00257-z. 38619655 10.1186/s41181-024-00257-z PMC11018728

[CR42] Network for Optimized Astatine labeled radiopharmaceuticals (NOAR) web. COST association; 2024.

[CR43] Organization ICA. Technical Instructions for the Safe Transport of Dangerous Goods by Air Montreal; 2023.

[CR44] O’Hara MJ, Krzysko AJ, Hamlin DK, Li Y, Dorman EF, Wilbur DS. Development of an autonomous solvent extraction system to isolate astatine-211 from dissolved cyclotron bombarded bismuth targets. Sci Rep. 2019;9:20318. 10.1038/s41598-019-56272-7. 31889075 10.1038/s41598-019-56272-7 PMC6937302

[CR45] PRISMAM Medical. Radionuclides/Our portfolio of medical radionuclides. 2025.

[CR46] Pozzi OR, Zalutsky MR. Radiopharmaceutical chemistry of targeted radiotherapeutics, part 3: alpha-particle-induced radiolytic effects on the chemical behaviour of At-211. J Nucl Med. 2007;48:1190–6. 10.2967/jnumed.106.038505. 17574991 10.2967/jnumed.106.038505

[CR47] Pozzi OR, Zalutsky MR. Radiopharmaceutical chemistry of targeted radiotherapeutics, part 4: strategies for (211)At labeling at high activities and radiation doses of (211)At alpha-particles. Nucl Med Biol. 2017;46:43–9. 10.1016/j.nucmedbio.2016.11.009. 28013121 10.1016/j.nucmedbio.2016.11.009 PMC5285439

[CR48] Prismap Medical Radionuclides. SCIPROM; 2025.

[CR49] Rabiei M, Asadi M, Yousefnia H. Astatine-211 Radiopharmaceuticals; Status, Trends, and the future. Curr Radiopharm. 2024;17:7–13. 10.2174/0118744710262325231025075638. 37937552 10.2174/0118744710262325231025075638

[CR50] Radchenko V, Morgenstern A, Jalilian AR, Ramogida CF, Cutler C, Duchemin C, et al. Production and supply of alpha-Particle-Emitting radionuclides for targeted alpha-Therapy. J Nucl Med. 2021;62:1495–503. 10.2967/jnumed.120.261016. 34301779 10.2967/jnumed.120.261016 PMC8612335

[CR51] Schlyer DJ, Ferrieri RA. Current status of solid state target technologies. IAEA: International Atomic Energy Agency, Vienna (Austria); 2000.

[CR52] Schultz MK, Hammond M, Cessna JT, Plascjak P, Norman B, Szajek L, et al. Assessing the 210At impurity in the production of 211At for radiotherapy by 210Po analysis via isotope Dilution alpha spectrometry. Appl Radiat Isot. 2006;64:1365–9. 10.1016/j.apradiso.2006.02.046. 16563782 10.1016/j.apradiso.2006.02.046

[CR53] Sevenois MBC, Miller BW, Jensen HJ, D’Huyvetter M, Covens P. Optimized cyclotron production of at: the challenge of Po-characterization. Radiat Phys Chem. 2023;212:ARTN111155.

[CR54] Tereshatov EE, Burns JD, Schultz SJ, Green BD, Picayo GA, McCann LA, et al. Ion exchange behavior of Astatine and bismuth. New J Chem. 2023;47:12037–47. 10.1039/d3nj01316b.

[CR55] Thera4Care/. Theranostics ecosystem for personalised care. Innovative Health Initiative; 2025.

[CR56] Tosato M, Favaretto C, Kleynhans J, Burgoyne AR, Gestin JF, van der Meulen NP, et al. Alpha atlas: mapping global production of alpha-emitting radionuclides for targeted alpha therapy. Nucl Med Biol. 2025;142–143:108990. 10.1016/j.nucmedbio.2024.108990. 39809026 10.1016/j.nucmedbio.2024.108990

[CR57] Vaidyanathan G, Zalutsky MR. Astatine radiopharmaceuticals: prospects and problems. Curr Radiopharm. 2008;1:177. 20150978 10.2174/1874471010801030177 PMC2818997

[CR58] Vanermen M, Ligeour M, Oliveira MC, Gestin JF, Elvas F, Navarro L, et al. Astatine-211 radiolabelling chemistry: from basics to advanced biological applications. EJNMMI Radiopharm Chem. 2024;9:69. 10.1186/s41181-024-00298-4. 39365487 10.1186/s41181-024-00298-4 PMC11452365

[CR59] Vretenar ALaLN M. Design of a helium ion linear accelerator for, astatine production. 16th Internationa Particle Accelartaor Conference Taipei Taiwan: JACoW; 2025. pp. 1067-70.

[CR60] Watabe T, Hatano K, Naka S, Sasaki H, Kamiya T, Shirakami Y, et al. First-in-human SPECT/CT imaging of [(211)At]PSMA-5: targeted alpha therapy in a patient with refractory prostate cancer. Eur J Nucl Med Mol Imaging. 2025;52:2253–5. 10.1007/s00259-024-07017-w. 39688698 10.1007/s00259-024-07017-w PMC12119378

[CR61] Watabe T, Mukai K, Naka S, Sasaki H, Kamiya T, Fukuhara A, et al. Phase I investigator-initiated clinical trial of targeted alpha therapy using [At]NaAt for refractory thyroid cancer (Alpha-T1 trial). J Nucl Med. 2025;66:251278.

[CR62] Watabe T, Mukai K, Naka S, Sasaki H, Kamiya T, Hayakawa T, et al. First-in-Human study of [(211)At]NaAt as targeted alpha-Therapy in patients with Radioiodine-Refractory thyroid cancer (Alpha-T1 Trial). J Nucl Med. 2025. 10.2967/jnumed.125.270810. 40998730 10.2967/jnumed.125.270810 PMC12676666

[CR63] Wilbur DS. Overcoming the Obstacles to clinical evaluation of (211)At-labeled radiopharmaceuticals. J Nucl Med. 2001;42:1516–8. 11585866

[CR64] Woen DH, Eiroa-Lledo C, Akin AC, Anderson NH, Bennett KT, Birnbaum ER, et al. A Solid-State support for separating Astatine-211 from bismuth. Inorg Chem. 2020;59:6137–46. 10.1021/acs.inorgchem.0c00221. 32302134 10.1021/acs.inorgchem.0c00221

[CR65] World Astatine. Community expand acces to cancerfighting At-211. Web: Nuclear Newswire; 2023.

[CR66] Zalutsky MR, Pruszynski M. Astatine-211: production and availability. Curr Radiopharm. 2011;4:177–85. 22201707 10.2174/1874471011104030177 PMC3503149

[CR67] Zalutsky MR, Reardon DA, Akabani G, Coleman E, Friedman AH, Friedman HS, et al. Clinical experience with alpha-particle-emitting At-211: treatment of recurrent brain tumor patients with At-211-labeled chimeric antitenascin monoclonal antibody 81C6. J Nucl Med. 2008;49:30–8. 10.2967/jnumed.107.046938. 18077533 10.2967/jnumed.107.046938 PMC2832604

[CR68] Zalutsky MR, Zhao XG, Alston KL, Bigner D. High-level production of alpha-particle-emitting At-211 and Preparation of At-211-Labeled antibodies for clinical use. J Nucl Med. 2001;42:1508–15. 11585865

[CR69] Zimmermann R. Is (211)At really happening? J Nucl Med. 2025;66:681–3. 10.2967/jnumed.125.269699. 40147851 10.2967/jnumed.125.269699

